# The Give-and-Take Interaction Between the Tumor Microenvironment and Immune Cells Regulating Tumor Progression and Repression

**DOI:** 10.3389/fimmu.2022.850856

**Published:** 2022-04-13

**Authors:** Simon Pernot, Serge Evrard, Abdel-Majid Khatib

**Affiliations:** ^1^ Reprograming Tumor Activity and Associated Microenvironment (RYTME), Bordeaux Institute of Oncology (BRIC)-Unité Mixte de Recherche (UMR) 1312 Inserm, Pessac, France; ^2^ Institut Bergonié, Bordeaux, France

**Keywords:** tumor microenvironment, neoplastic evolution, reciprocal interaction, metastatic dissemination, stromal cells secreted molecules, philosophy of cancer

## Abstract

A fundamental concern of the majority of cancer scientists is related to the identification of mechanisms involved in the evolution of neoplastic cells at the cellular and molecular level and how these processes are able to control cancer cells appearance and death. In addition to the genome contribution, such mechanisms involve reciprocal interactions between tumor cells and stromal cells within the tumor microenvironment (TME). Indeed, tumor cells survival and growth rely on dynamic properties controlling pro and anti-tumorigenic processes. The anti-tumorigenic function of the TME is mainly regulated by immune cells such as dendritic cells, natural killer cells, cytotoxic T cells and macrophages and normal fibroblasts. The pro-tumorigenic function is also mediated by other immune cells such as myeloid-derived suppressor cells, M2-tumor-associated macrophages (TAMs) and regulatory T (Treg) cells, as well as carcinoma-associated fibroblasts (CAFs), adipocytes (CAA) and endothelial cells. Several of these cells can show both, pro- and antitumorigenic activity. Here we highlight the importance of the reciprocal interactions between tumor cells and stromal cells in the self-centered behavior of cancer cells and how these complex cellular interactions control tumor progression and repression.

## Introduction

Cancer is a multi-causal and multi-level process categorized by heterogeneity of effects, mainly affecting various cellular functions that regulate neoplasia process and progression. Previously, cancer was considered as a disease related to the environment and endogenous factors, and lately molecular and genetic studies emerged in attempt to explain cancer and to understand its main mechanisms (1). It is now evident that cancer cells disrupt the rules and function of normal cells. Indeed, cancer cells divide and proliferate when is not necessary, do not die when is required, take advantage of the resources of other normal cells and perturb the harmony of the normal tissue environment. Furthermore, while the collaborating “normal” cells have limited proliferative capacity, tumor cells can resist to cell death and escape from the immune system. Furthermore, while normal cells generate and use biological signals and mediators essential for their functions and survival, cancer cells transform their surrounding normal cells to use more resources for themselves in order to grow and spread indefinitely in an egocentric manner. Thereby, cancer cells can be considered as newly modified normal cells that have stopped interacting normally with the other immediate cells. During these processes the transformed cells adapt malignant mechanisms to impose their control of the newly transforming microenvironment (2). However, although these cellular interactions participate in carcinogenesis, they can also repress tumor growth and evolution. Thereby, what are the elements that, once affected, are responsible for the self-centered behaviors of cancer cells? What are the mechanisms to be defined in order to explain why cancer can or cannot develop following these interactions? Why cancer cells and not normal cells that take advantage of this cellular interactions? In addition, it is also difficult to explain how certain primary tumors remain dormant unable to progress and form metastases while interacting with stromal cells? Finally, what are the factors that suddenly make these tumors to emerge and start to grow. In this review we address the importance of the major cellular interactions in the tumor microenvironment that control tumor cells and how these interactions influence the causality of cancer and neoplastic evolution. We consider the following:

### Dynamic Reciprocity

Denotes bidirectional communication in all kinds of cells, involving specifically the nucleus and cellular extracellular matrix (ECM) elements.

### Reciprocal Cellular Interaction

Bidirectional interaction between cells and their microenvironment. During this interaction, cells within a specific tissue express and produce a panel of signals/mediators to which other tissues and cells can react and reply. In turn, the responding cells produce distinct signals to which the signaling cells also respond. In this interaction all the involved cells are signaling and responding elements.

## Dynamic Reciprocity Model: From Homeostasis of Single Cell to Cancer Tissue

**Figure 1:**
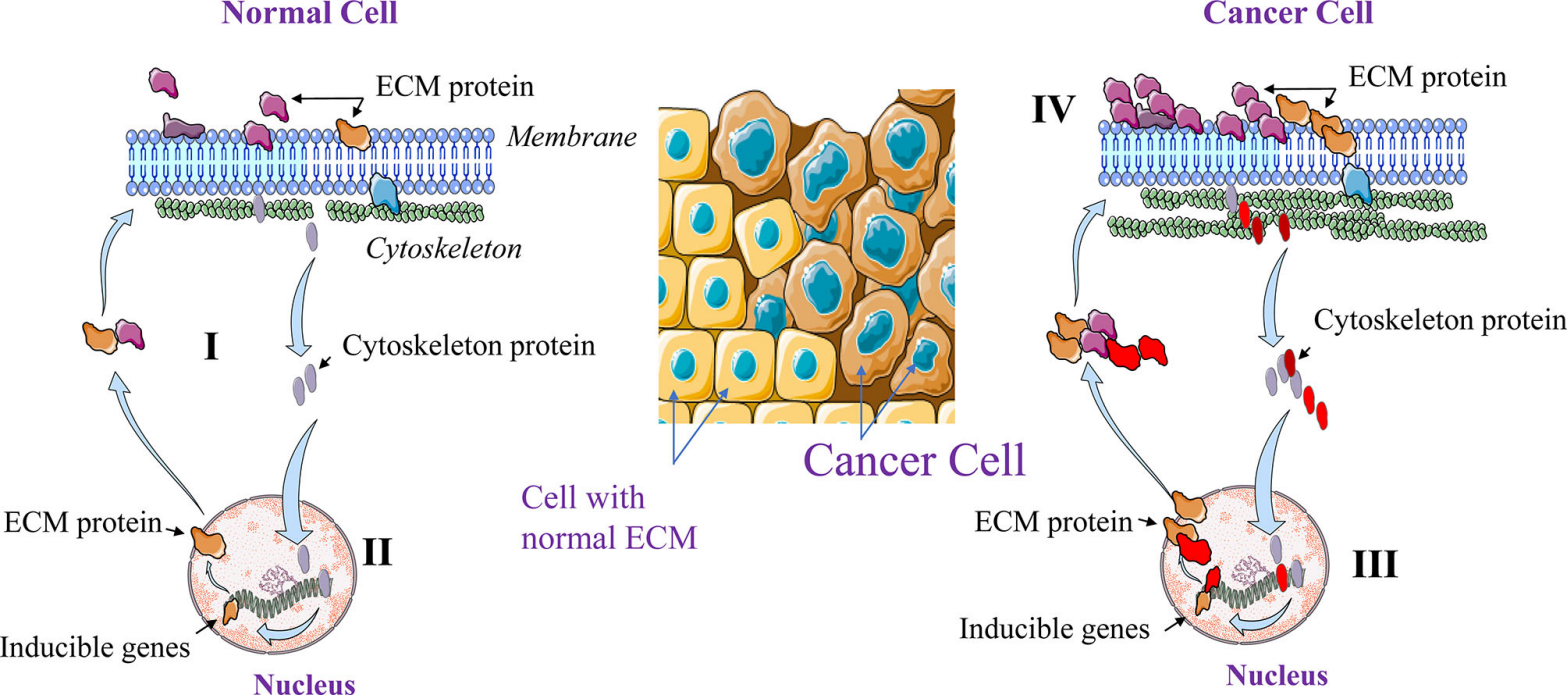
Dynamic Reciprocating Model (DRM). At the normal single cell level, the cytoskeleton and the nuclear matrix interact in a reciprocal manner (I). This interaction maintains normal cellular architecture and functions through ECM components expression and remolding. Activated cytoskeleton molecules mediate the activation of various nuclear promotors that in turn induce the expression of molecules including ECM components that control the cellular shape and structure (II). In cancer cells this regulation is altered leading to high cytoskeleton and ECM components expression (III) and activation (IV).

Normal tissue formation relies on wide range of cell types with specific function and complementary roles, and on secreted factors or mediators that mediate various effects on the developing tissue and its cellular interactions. However, deregulations at the single cell level may contribute to global cancer initiation, progression and dissemination. In 1982, Bissell et al. (3) and later in 2009, Xu et al. (4) introduced the Dynamic Reciprocity Model (DRM) to explain at the cell level how the cross talk between several cell constituents can be involved in carcinogenesis and metastasis. This model assumes that at the cell level some constituents such as the cytoskeleton and the nuclear matrix are involved in a reciprocal interaction with the ECM to maintain the normal architecture and the function of specific cell in a specific tissue or organ (**Figure 1**). Indeed, in addition to its role in the cell movement and shape design, through its intracellular molecules, the cytoskeleton is also involved in the regulation of various cellular functions at both, the cellular and genetic levels. Following their activation, various cytoskeleton molecules mediate through distinct and interconnected signals the activation of various nuclear promotors. In turn, the latter induce the expression of ECM proteins that control the cellular shape and structure. Interestingly, the cytoskeleton and ECM molecules are also regulatory factors of cell proliferation, motility and survival/apoptosis. Thereby we can speculate that cancer occurs when the dynamic and reciprocal interaction mediated by the cytoskeleton and nucleus is compromised or altered. Undeniably, the expression and the accumulation of ECM components such as collagen, proteoglycans and their remodeling enzymes (MMPs, Urokinases) are strictly regulated in order to maintain normal cellular functions and shape. Dysregulation in the expression of these ECM molecules and alteration of the proteolytic activity of the ECM enzymes affect the tissue microenvironment, leading to tumorigenesis and tumor cell dissemination (**Figure 1**). We can assume that the cytoskeleton is therefore essential in both cellular and ECM regulation, although much work remains to be done to define the mechanisms involved.

## Reciprocal Cellular Interaction and Tumor Progression

Tumors are considered as new developing organs that contain both malignant cells and wide range of non-malignant cells that lost their ability to continuously preserve tissue homeostasis and architecture. The main non-malignant cells that participate in the constitution of the tumor tissue are immune cells (e.g. macrophages/TAMs, NK, and T cells), fibroblasts/CAFs, endothelial cells and adipocytes/CAAs (5). The communication between cancer cells and these non-malignant cells seemed to control the outcome and the evolution of the developed tumors. In normal tissues the cellular interactions can be mediated through physical communication involving receptors and/or various ECM constituents and ECM-imbedded enzymes and through diverse range of mediators produced by the involved interacting cells (5, 6). These include growth factors, chemokines and cytokines and other mediators of which the expression and production are strictly regulated (7). The proteolytic activation of these molecules or expression was reported to be also required for cancer cell interaction with the TME (8–11). Thereby, the disturbance of the tissue homeostasis generates changes in the activity and the function of the communicating cells. In turn these changes affect cell proliferation, migration and survival leading to tumor progression or repression (7). In this section we will address the major cell-cell interactions within the tumor microenvironment involving cancer cells and non-malignant cells and their role in the neoplastic evolution and the outcome of tumor progression and repression.

## Cancer Cells and Immune Cells Interaction

By infiltrating tumors, immune cells mediate cytotoxic effect on cancer cells. Although the hypothesis about the host defense that represses cancer cells growth were formulated in 1909 by Paul Ehrlich (12), the concept of immunological surveillance was introduced by Frank MacFarlane Burnet in early 70s (6). Indeed, based on the hypothesis that neo-antigens expression on tumor cells are able to induce immunological events against cancer cells he formulated the immune surveillance theory (6). He wrote that: “It is by no means inconceivable that small accumulation of tumor cells may develop and because of their possession of new antigenic potentialities provoke an effective immunological reaction with regression of the tumor and no clinical hint of its existence” (6). However, the efficacy of the tumor-infiltrating immune cells can be evaded by cancer cells using complex mechanisms where various immune cells are driven to participate in tumor initiation and progression.

The interaction of immune cells and cancer cells are mainly summarized in three major phases (13). During all these steps, cancer cells and immune cells use various mediators and interactors that determine tumor growth or repression. These include: **1**-the cancer cells clearance phase or elimination, where the immune cells are able to eradicate the newly transformed cells, **2**-the equilibrium phase, that corresponds to a balance between the eradicated and the newly formed cancer cells due to the maintained activity of immune cells (14). In this phase the tumor cells are considered as dormant. The last step is the escape phase characterized by the accumulation of tumor-cell variant subpopulations (or clones) occurred during the equilibrium phase (15). This process is the direct consequence of the heterogeneity of the transformed cells that subsequently results in the development of cellular mechanisms allowing immune cells escape or suppression (16). The generated cancer cell clones increase their ability to grow and proliferate in an immunocompetent environment.

## Elimination Phase

**Figure 2:**
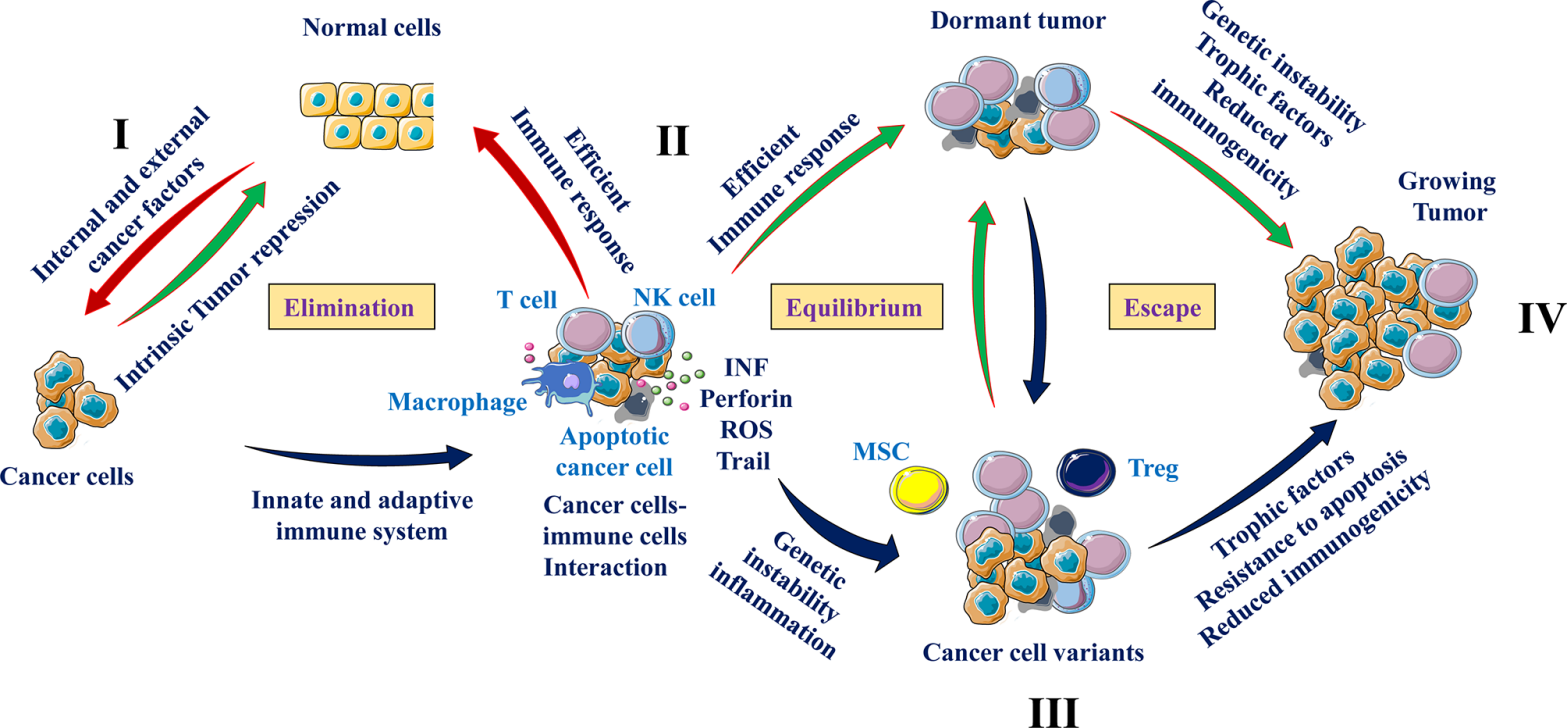
Cancer cells and immune cells communication. From immune surveillance to immune escape. Exposure to internal and/or external risk factors induced cell transformation (I) of which the development is repressed by intrinsic tumor suppressing mechanisms (e. g. tumor suppressor genes and apoptosis) (II). During the elimination phase, immune cells such as natural killer cells (NK), and T cells are able to recognize (e.g. through presence of NKG2D ligand) and eliminate tumor cells. The equilibrium phase involves the continuous elimination of tumor cells by immune cells secreting cytotoxic agents (INF, perforin and others) and the accumulation of resistant cancer cell variants (III). At the escape phase, as a result of heterogeneity, tumor cells that are less immunogenic are able to escape immunosurveillance and secrete cytokines and chemokines that recruit immunosuppressive cells (Tregs, MSCs and MDSCs), which suppress the antitumor immune responses through different pathways including T cell and NK cell activity repression (IV).

In this cellular cross talk, the activity of the immune cells is optimal and able to stop progression of tumor growth (**Figure 2**). Indeed, under normal conditions, immune cells mediate their cytotoxic activity only following the acquisition of the transformed phenotype by cancer cells (16). The elimination of cancer cells by the immune cells involves both the innate (general and rapid) and the adaptive (specialized) immune systems (17). During these processes, immune cells such as T cells and macrophages are able to distinguish tumor cells from normal cells through expression of specific molecules such as the ligands for NKG2D on tumor cells. Throughout cell transformation, the release of various proinflammatory molecules and chemokines by tumor cells is enough to activate the innate immune system. Following cancer cells recognition, the immune cells secrete various molecules such as IFN-γ and perforin that eliminate the emerging tumor cells. These processes are then gradually increased leading to high production of cytokines and chemokines that permit the recruitment of more immune cells. In turn, the activated immune cells participate in the accumulation of cytotoxic products such as perforin, reactive oxygen or TRAIL (16). Thereby, this positive loop of immune cells recruitment and activation enhances the generation of tumor antigens of dead tumor cells. Subsequently, the generated antigens induce the activation of the adaptive immune system that in turn participate in the ongoing cancer cells elimination process. Thus, this phase of cancer cells and immune cells interaction is an uninterrupted mechanism where the newly formed cancer cells expressing specific markers and cytokines are identified by immune cells and eliminated.

## Equilibrium Phase

The tumor cells that have survived the elimination phase enter into a dynamic equilibrium with their interacting immune cells. This phase is the longest of the three phases of the cancer cells and immune cells interaction and may persist for many years (dormant tumors) (16). The T lymphocytes are the main immune cells involved in the equilibrium phase where they secure the preservation of this cancer cells and immune cells communication. Cytokines like IFN and TNF were also found to be involved during these interactions (18). In addition, IFN was found to be a key player in the progression from the elimination phase to equilibrium phase through its selective immunoediting pressure (16). Despite the elimination of abundant emerging tumor cells, several tumor cell variants with different mutations may resist and survive to the activated immune cells. During this period, the heterogeneity and genetic instability of cancer cells that subsist the elimination phase are the main causes. Of these processes the nucleotide-excision repair instability (NIN), microsatellite instability (MIN), and the chromosomal instability (CIN) (19, 20) are the major genetic instability that were found to provide tumor cells variants with reduced immunogenicity and/or ability to grow in a highly immunocompetent environment. The altered environment with accumulated cytokines and hypoxia also favors immunosuppression leading to the escape phase (**Figure 2**).

## Escape Phase

Tumor cell variants derived from the equilibrium phase carry various genetic changes that confer cancer cells resistance to immune detection and elimination, allowing the tumors to expand and grow. At this phase, cancer cells use various strategies to avoid the immunosurveillance of the innate and/or adaptive immune system. They can repress the anti-tumoral immune responses through the production of immunosuppressive cytokines such as TGF-β and IL-10 or through the recruitment of T cells with immunosuppressive activities such as the regulatory T cells, MSCs and MDSCs (14, 21) (**Figure 2**). The alterations that occur on tumor cells can also affect tumor recognition by immune cells. These include altered cell surface antigen expression, loss of MHC elements (22), liberation of NKG2D ligands (23), and resistance to IFN-γ effect (24). The expression of aberrant antigens on tumor cells also affects the anti-tumoral response by inhibiting the proliferative response of immune cells (25). Production of MSCs with potent immunosuppressive function may also contribute to inactivation of T cells through various mediators such as nitric oxide (NO) that limit the proliferation and mediate apoptosis of T cells (25, 26).

## Crosstalk Between Fibroblasts and Cancer Cells

**Figure 3:**
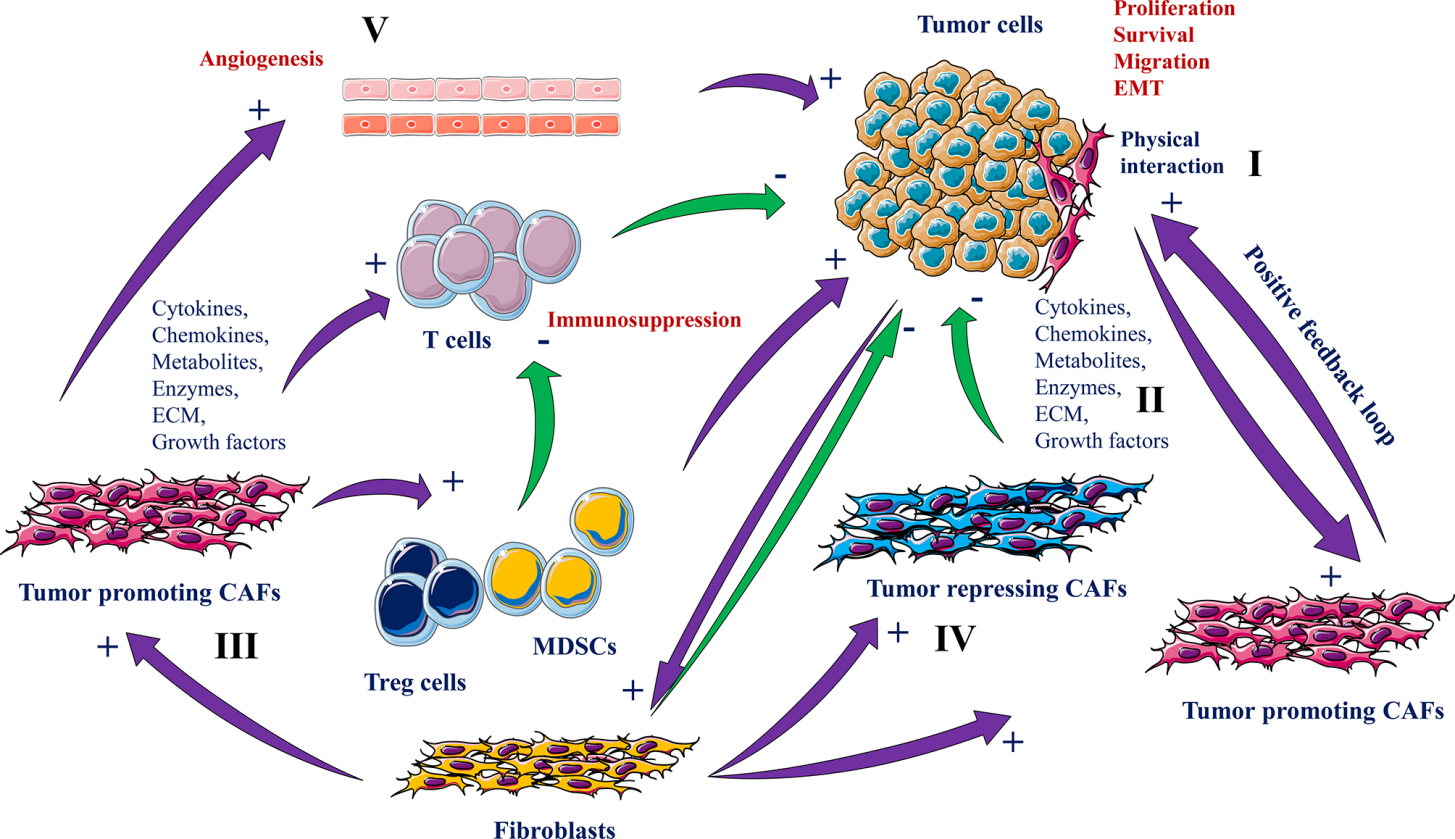
Roles of CAFs and cancer cells interaction in tumorigenesis. CAFs derived from normal fibroblasts interact with tumor cells physically (I) or through secreted molecules and ECM remodeling (II) that either promotes (III) or suppresses tumorigenesis (IV). While the tumor-promoting CAFs participate in tumor cell growth, survival and migration, angiogenesis (V), EMT and immune cell activity repression, the tumor repressing CAFs and normal fibroblasts inhibit these processes.

In normal tissues, fibroblasts are responsible for tissue integrity (27). These cells can reply to tissue damage by their differentiation to myofibroblasts that in turn coordinate the wound healing process and the repair of the damaged tissue. These biological functions are mediated by ECM synthesis and remodeling and through the permanent fibroblasts interaction with immune cells (28). Fibroblasts within the tumor microenvironment that exhibit a cancer-associated phenotype are denoted as “cancer-associated fibroblasts” or CAFs (29). During tumor initiation and progression, the physiological functions of normal fibroblasts are exaggerated in CAFs which agrees with the image of tumors as “wounds unable to heal” (30). CAFs produce and release various cytokines, chemokines, metabolites, enzymes and ECM molecules that induce cancer cell proliferation, and migration and other processes that support tumor growth and the malignant phenotype of tumor cells. These include angiogenesis and Epithelial-Mesenchymal Transition (EMT) (29–31). However, these molecules when produced by CAFs can also repress tumor growth and progression (29, 32, 33) (**Figure 3**).

## Pro-Tumorigenic Function of CAFs

The secreted molecules by the CAFs are the main driver of their pro-tumorigenic function. Of the mediators involved in the cross talk between CAFs and cancer cells are various chemokines (e.g. CXCL12, CCL7) (34, 35), growth factors (TGFβs, FGFs and HGF) and several ECM proteins (collagens and proteoglycans) (36–40). These molecules induce tumor progression by directly increasing cancer cell survival, proliferation, stemness, and the acquisition of the metastatic phenotype. On the other hand, cancer cells also secrete similar molecules that stimulate CAFs activity and thereby leading to the generation of a set of signaling activities converging to the promotion of tumor progression (41, 42). The cancer cells and CAF-derived molecules can also function in a positive feedback loop to increase and maintain CAFs activation (28). For example, both CAFs and cancer cells are able to secrete LIF to activate CAFs and their ECM remodeling that favors CAFs migration together with cancer cells in a combined manner (43). CAFs are also involved in the induction and enhancement of angiogenesis through their ability to produce and secrete various proangiogenic factors such as VEGF (44). Similarly, through their ability to secrete inflammatory molecules such as IL-1, IL-6 and TNFα, CAFs participate in inflammation-mediated tumor progression (45). Various of the CAFs secreted molecules participate as well in the generation of an immunosuppressive microenvironment that suppresses the immune system activity and favors tumor escape and progression (29, 45, 46). Furthermore, CAFs contribute to tumor progression through their role in the regulation of the metabolic activity within the tumor microenvironment (29, 45, 46) (**Figure 3**).

By direct contact with cancer cells CAFs can also facilitate metastasis. During this physical interaction CAFs were reported to exert mechanical force on cancer cells to allow cooperative invasion (47). This Force is mediated by junction between E-cadherin expressed by cancer cells and N-cadherin expressed by CAFs. Physical interaction between CAFs and cancer cells can also trigger activation of signaling pathways involved in tumor cell invasion (47). Alteration of this cellular connection abolishes the CAFs migration during cancer cells invasion (47). Similarly, CAFs were found to mediate invasion of cancer cells through generation of migratory tracks in the ECM matrix where cancer cells follow CAFs to mediate cooperative invasion (48). The role of CAFs in the metastatic phenotype of tumor cells was also linked to EMT. Cancer cells undergoing EMT lose their ability to mediate cell-cell interactions and were found to have enhanced secretion of IL-6 (29). In turn, this cytokine activates CAFs to secrete several MMPs that further increase the EMT phenotype in cancer cells and subsequently facilitating cancer cells dissemination and metastases formation (29).

## Antitumor Function of CAFs

Compared to the pro-tumorigenic function of CAFs, several secreted CAF mediators can exert antitumor functions such as IFNγ. This factor promotes an anti-tumoral immune pressure against cancer cells through the recruitment of immune cells and their secreted molecules (29, 45, 49–51). Thus, the CAFs seemed to have a dual role probably linked to their heterogeneous populations and the nature of their secreted mediators (**Figure 3**).

## Endothelial Cells and Cancer Cells Interaction

The importance of endothelial cells in the context of cancer has been widely explored. Already in 1945, it was reported that inoculated tumors in mice are able to recruit vessels directly from surrounding tumor area (52). Lately, in the early 70s, a factor with activating effect on the formation of new tumor vascular vessels was isolated from the related tumors (53, 54). Subsequently, the appearance of new blood vessels formation was designed as a predictable property of various cancer cell types. This event was described by Judah Folkman as the initial sign that a group of cell populations has become dedicated to neoplasia (53). Cancer cells need nutrients and oxygen derived from their close blood vessels in order to survive and proliferate. Tumors attain their vascularization by inducing the formation of new vascular vessels, a process known as tumor angiogenesis, or by using pre-existing vasculature, a phenomenon described as vascular co-option (55).

**Figure 4:**
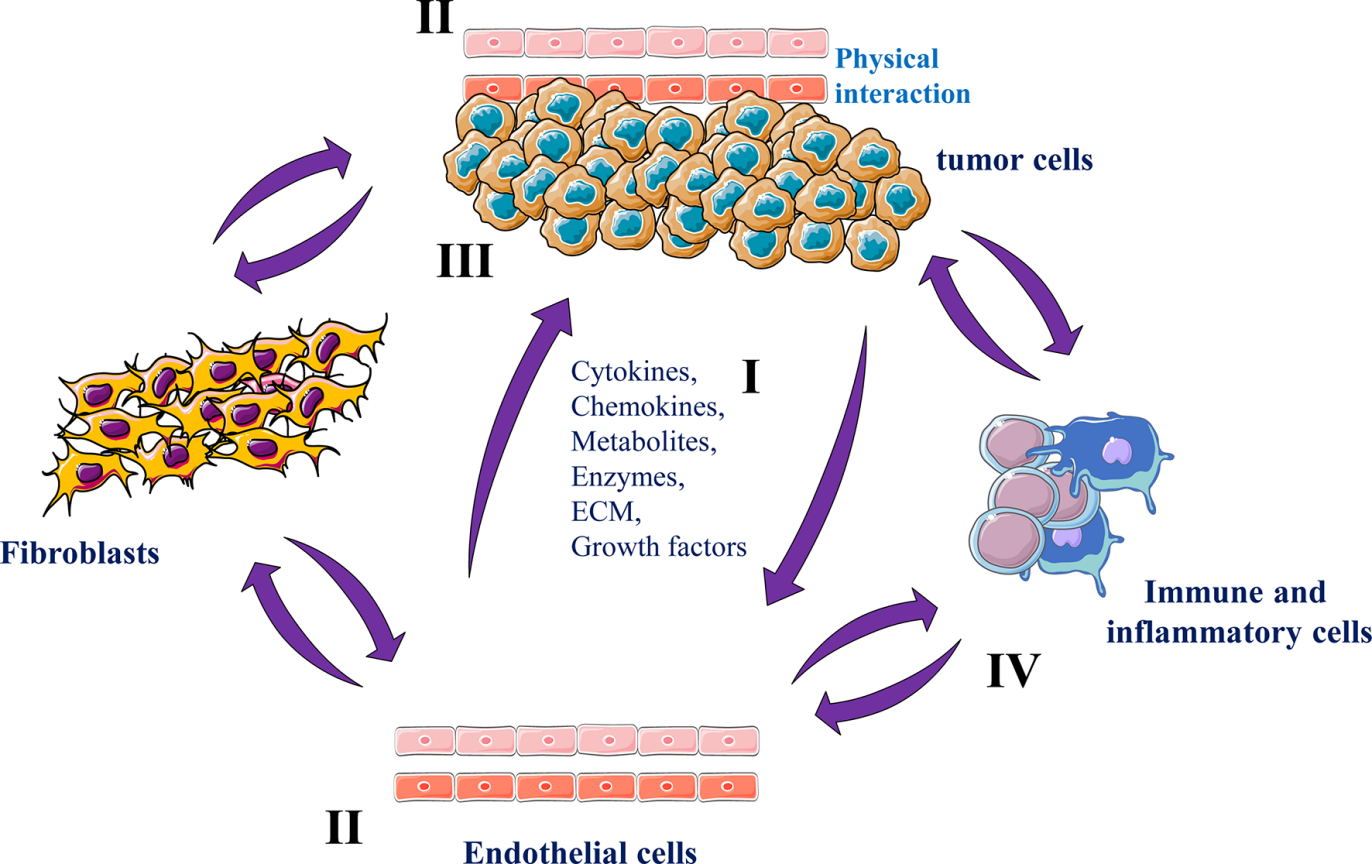
Endothelial cells and cancer cells interactions Tumor endothelial cells and tumor cells secrete various molecules and ECM components in the tumor microenvironment (I) that mediate tumor angiogenesis (II) and growth (III). Tumor endothelial cells can also reciprocally interact with various stromal cells that in turn participate in tumor progression and angiogenesis and (IV).

The onset of malignancy requires also reciprocal interactions between endothelial cells and tumor cells (56). Tumor neovascularization can also affect the microenvironment of the growing tumor and cause tumor immunosuppression by recruiting immunosuppressive cells, and inhibiting cytotoxic T cell activity through angiogenic factors (57). In turn, the activated tumor microenvironment releases a large number of factors that promote tumor angiogenesis, establishing a tumor growth-promoting cycle (58) (**Figure 4**). In addition, the formed tumor vessels are immature with high permeability and hypoxia that further facilitates tumor growth and metastasis (59).

In normal conditions, endothelial cells in adult and mature organs are inactive and dormant cells. These cells can be activated to migrate and proliferate by pro-angiogenic factors (60). This angiogenic switch participate in the activation of dormant tumors that become more proliferative and acquire more aggressive and metastatic phenotypes (56). Compared to normal endothelial cells, tumor endothelial cells are morphologically and functionally different and altered (61). Within the tumors these cells acquire new capacities able to provide tumors with proliferative and survival benefits.

The interactions between tumor cells and endothelial cells are complex involving various reciprocal signaling mechanisms and physical interactions. However, products derived from both tumor cells and endothelial cells have been implicated in these interactions (62). While tumor cells mediate tumor angiogenesis by secreting soluble factors such as VEGF and PDGF that enhance endothelial cells proliferation, migration and vessels formation (62, 63), endothelial cells secrete wide range of pro-inflammatory cytokines and chemokines (e.g. IL-1β, IL-6, TNF-α, CXCL1 and CCL2 (64, 65) that stimulate cancer cells and non-malignant cells within the tumor microenvironment, important process in potentiating inflammatory responses and immune cells activity regulation. Endothelial cells are also capable of producing various growth factors including PDGF, VEGF and FGF (66) that play a critical growth role during tumor progression and act as survival factors, avoiding the apoptotic death of both cancer and endothelial cells (66–68) (**Figure 4**).

To date, there is established agreement that the arrest of circulating cancer cells due to their physical interaction with endothelial cells is essential for their migration from the blood stream and successive growth into metastatic lesions (69, 70). Specificity in the site of cancer cell arrest has been identified as one factor causative to the organ-specific metastatic patterns (69, 70). This process involves specific interactions between the surface of circulating cancer cells and endothelial cells through various adhesion molecules such as selectins, integrins, cadherins, and immunoglobulins (69, 70). Some of these molecules are expressed constitutively and appear to have organ specificity in their distribution. Others are inducible and under the influence of environmental signals, such as cytokines (69, 70). The induction of these molecules on endothelial cells was reported to be link the metastatic capacity of the interacting tumor cells (69, 70). Indeed, compared with low metastatic or nonmetastatic colon cancer cells, only the arrest of highly metastatic ones in the hepatic circulation was found to mediate this cascade of events. Indeed, the physical interaction of tumor cells with liver endothelial cells induces a rapid release of several cytokines such as TNF-α and IL-1 that in turn stimulate the expression of E-selectin and other adhesion molecules on hepatic endothelial cells, leading to enhanced tumor cell adhesion in the liver (69, 70). Subsequently, the growth of the colonizing metastatic cells is sustained by the overexpression and/or increased activity of other molecules such as growth factors and cytokines (69, 70). The expression and the activity of these molecules were found to require the involvement of the convertases for the mediation of their functions (71–76). Indeed, the inhibition of these enzymes in metastatic cancer cells was reported to inhibit E-selectin expression and their adhesion on endothelial cells leading to repression of metastases formation in the liver of tumor cells-inoculated mice (8, 77). Further analysis, revealed that the convertases found in cancer cells contribute to metastasis by enhancing the level of active cytokines (TNF, Il-1) involved in the first step of cancer cell and endothelial cell interaction required for liver colonization by tumor cells (8).

## Myeloid Derived Suppressor Cells and Cancer Cell Interaction

Myeloid derived suppressor cells (MDSC) are heterogeneous populations of immune cells that were initially identified in 1970 (78). These cells are characterized by their suppressive effects on immune cells and found to expand during tumor progression. MDSCs can mediate their immunosuppressive function alone or through their interaction with other myeloid cells including tumor-associated neutrophils (TANs), tumor-associated macrophages (TAMs), and regulatory dendritic cells (79).

MDSCs are mainly divided into two important groups: the monocytic MDSCs (m-MDSCs) and granulocytic MDSCs (g-MDSCs) groups. The expansion of MDSCs is induced by various released factors produced by tumor cells, stromal cells, T-cells or macrophages. These include cytokines, prostaglandin E2 (PGE2), MMPs, chemokine and growth factor (80). Expression of inducible nitric oxide synthase (iNOS) and arginase I was reported to be involved in MDSCs-induced suppression of the proliferation and the cytotoxic function of T cells (81). MDSCs can also indirectly suppress anti-tumor immunity, through inhibition of TILs and the generation of regulatory T-cells (Tregs) in the tumor microenvironment following their production of TGF-β and several cytokines (82). MDSCs also contribute to tumor progression *via* various other mechanisms including their ability to mediate angiogenesis (83) and epithelial–mesenchymal transition (EMT) (84). These cells can also mediate cancer cell invasion by secreting matrix metalloproteinase-9 (MMP9) (85) (**Figure 3**).

## Tumor-Associated Macrophages (TAMs) and Cancer Cells Communication

In addition to cancer-associated fibroblasts (CAFs) and tumor vascular endothelial cells the tumor-associated macrophages (TAMs) are also important constituents of the tumor microenvironment where they play a key role in the interaction of cancer cells with the immune components of the tumor microenvironment (86). In early 70s, TAMs were reported to promote tumor growth (87) and only in the last decades, TAMs were divided into two broad categories: the pro-inflammatory M1 macrophages with antitumor properties and anti-inflammatory M2 macrophages with tumor-promoting functions (87). In the majority of tumors, the TAMs are mainly M2 macrophages actively involved in tumor growth and dissemination (88). However, TAMs were found to mediate both M1 and M2 macrophage functions (87). Usually TAMs derive from circulating monocytes and tissue-resident macrophages (89) and their effect on tumor progression and metastasis is linked to the nature of tumor microenvironment, the tumor type and their localization within the tumor (89). By secreting molecules including EGF, FGF and TGFβ the TAMs directly affect cancer cell proliferation (87, 90). Similarly, through the upregulated secretion of various pro-angiogenic factors, such VEGF-A, TNFα, FGF and others, TAMs promote vascular vessel formation and through production of molecules such as VEGF-C and VEGF-D the TAMs induce also the formation of lymphatic vessels (90). The release of several enzymes such as plasmin, and MMPs, TAMs also participate in tumor cells invasion and metastasis (91, 92). TAMs can also promote metastasis through the release of exosomes containing various miRNA and oncogenic proteins (91).

**Figure 5:**
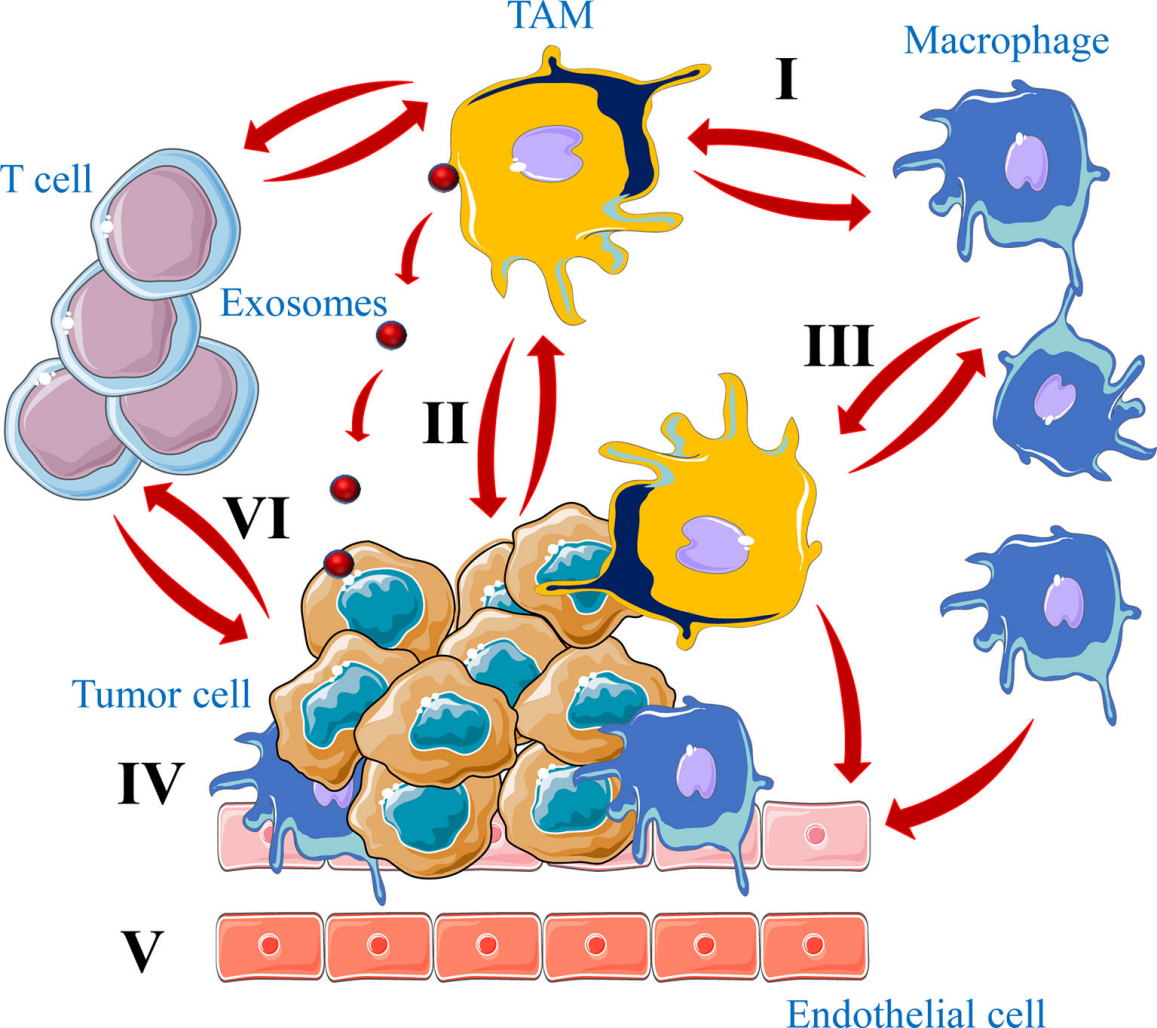
Crosstalk between tumor cells and TAMs in tumors. Macrophages have the function of killing tumor cells (I). However, a large number of TAMs infiltrated in the tumor microenvironment not only kill tumor cells, but also promote tumor growth and metastasis (II). In the tumor microenvironment tumor cells secrete a large number of chemokines and cytokines to recruit macrophages into tumors and regulate their signaling pathway to induce M2 macrophages formation (III). Finally, TAMs differentiated into M2 type maintain tumor growth by promoting tumor cell growth (IV), angiogenesis (V) and immune cells repression (VI), through physical interaction, secreted molecules or exosomes.

For their reciprocal interaction with TAMs, tumor cells were found to secrete some signal molecules and cytokines such as CSF1 and IL-6 required for TAMs activation. The expression of these cytokines by tumor cells is associated with the infiltration of TAMs in the growing tumors (93). The release of these cytokines by cancer cells induce macrophages to secreted the same cytokines (94–96) that further mediate TAMs recruitment to the newly developed tumor, indicating that the interaction between tumor cells and TAMs forms a secretory rotation that promotes the recruitment of macrophages in tumors and subsequently tumor growth and progression (**Figure 5**).

By secreting wide range of cytokines and chemokines, such as CCL3, CCL5, CCL22 and TGFβ, TAMs participate also in the recruitment of regulatory T cells (Treg) to the tumor microenvironment and suppress cytotoxic T cell functions (92, 97, 98). TAMs ablation was found to block Treg cell recruitment and inhibits tumor growth by lowering the CCL20 level in mice model (99). In addition, TAMs can inhibit cytotoxic T-cell proliferation through several mechanism such as the secretion of IL-10, prostaglandins, TGF-β and reactive oxygen species (ROS) (100, 101). Furthermore, various molecular mechanisms used by tumor cells during their interaction with macrophages mediate tumor immune escape. For example, like in activated T cells, TAMs express the programmed cell death ligand 1 (PD-L1), a ligand of the immune checkpoint receptor programmed cell death protein 1 (PD-1) that contributes to the generation of an immune-suppressive tumor microenvironment. This by repressing normal function of macrophages including cytokine release, antigen presentation and phagocytosis (102). Accordingly, PD-1 expression by TAMs increases with tumor progression and the blockade of PD-1 and PDL-1 interaction was found to reduce tumor growth in mice models (103). Similarly, to escape immune system tumor cells express on their surface CD47 that functions as an inhibitor of phagocytosis following its interaction with the signal-regulatory protein alpha (SIRPα) expressed on macrophages cell surface (104). Indeed, SIRPα/CD47 pathway is referred to as the “do-not-eat-me” signal and tumor cells with CD47 expression can be recognized as self-normal cells and escape phagocytosis (105, 106) (**Figure 5**).

M1-type macrophages are able to distinguish tumor cells from normal cells and kill tumor cells. M1-type macrophages use mainly two different mechanism to directly eliminate tumor cells: **1**-direct cytotoxic action that involves the release of multiple cytotoxic molecules such as ROS and NO (107) and **2-** through antibody-dependent cellular cytotoxicity (ADCC) that directly target tumor cells (108). Other indirect mechanisms were also reported to be involved in tumor cells elimination by M1-type macrophages such as the expression of cytokines (IFN-γ, IL-1, and IL-6) that activate the cytotoxic Th1 cells leading to an antitumoral immune response activation (109, 110).

## Adipocytes and Cancer Cells Interaction

**Figure 6:**
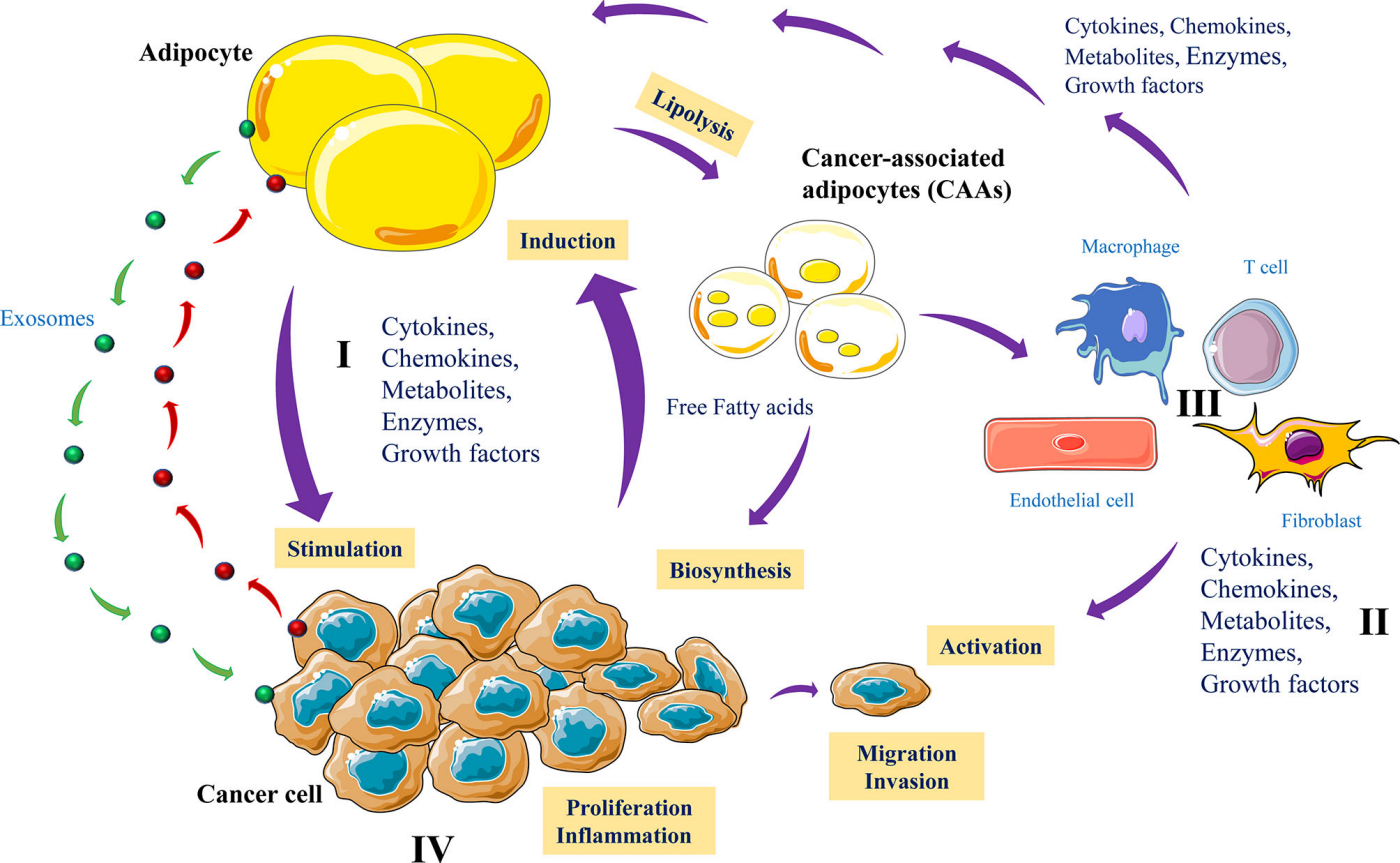
Adipocytes and cancer cells communication. Cancer cells produce various mediators including exosomes to reprogram metabolic activity of adipocytes (I). In turn, cancer-associated adipocytes (CAAs) produce growth factors, cytokines, MMPs and release exosomes that directly affect tumor cell growth and invasion (II). Lipolysis activated by tumor cells reduced lipid droplets in adipocytes and the CAA products such as free fatty acids are used as energy fuel molecules to promote tumor growth, angiogenesis, inflammation and metastasis (III). In parallel, the adipocyte-derived factors activate various nontumor cells to control the malignant and metastatic phenotype of tumor cells (IV).

Increased use of lipids by cancer cells is a hallmark of cancer progression and dissemination (2) and in various cancers, the presence of adipocytes can be largely predominant in the tumor tissues. where they mediate various reciprocal interactions with cancer cells (111). This cross-talk can be either by means of physical interactions or through secreted factors (**Figure 6**). Thereby cancer cells mediate reprogramming of adipocyte metabolic activity that acquire a cancer-associated adipocyte (CAAs) phenotype (112). During this interaction adipocytes by undergoing lipolysis serve as a source of lipids for cancer cells, used to increase their proliferation and invasiveness (113–115). Accordingly, adipocytes close to cancer cells or physically interacting with tumor cells were found to have reduced lipid droplets compared to adipocytes distant from the tumors (111, 116). Similarly, adipocytes co-cultured with cancer cells were found to lose their lipid droplets (116). Indeed, tumor cells are able to synthesize most fatty acids (FAs), essential cellular process that allow the conversion of nutrients into metabolites used for energy filling, protein synthesis and the generation of signaling molecules involved in various biological functions (117). However, when endogenous lipogenesis becomes insufficient for tumor progression and survival, cancer cells take advantage of the cancer-associated adipocytes to acquire additional extracellular FAs (118) that participate in their more malignant and metastatic phenotype. Cancer cells use various mechanisms and mediators to induce adipocyte lipolysis. These include extracellular vesicles that transfer cytokines such as IL-6 (119) leading to activation of NFκB signaling and inflammatory phenotype (120). In turn, activated adipocytes secrete chemokines (CCL2, and CCL5), cytokines (IL-1β, IL-6, TNF-α), and angiogenic factors (VEGF), all required for the promotion of tumor growth, angiogenesis and metastasis (115, 121, 122). Interestingly, adipocytes like tumor cells also secrete exosomes that are used by cancer cells to favor their migration and invasion (**Figure 6**).

Recent studies have shown that exosomes contribute to tumor invasion *via* their influence on the intercellular space (123) and contain high levels of several ECM proteases (MMP3 and MMP-9) involved in invasion (123). These proteases are also involved in lipid metabolism, and modulate cancer cell migration through metabolic reprogramming and mitochondrial activity (123). Recent proteomic analysis revealed that adipocyte exosomes contain more than 1000 different proteins. Of these, several are involved in protein turnover, metabolic activity and tissue repair (124). These studies indicate that adipocyte exosomes could promote tumorigenesis or drive cancer cells towards a more aggressive phenotype by the storage of various oncogenic molecules delivered to cancer cells. Activated adipocytes were also found to mediate immune repression following the expression of PD-L1 (104) and found to regulate anti-tumor immune response through NK cell activation (103).

## Conclusions

In tumors, cells lose their normal behavior such as their ability to differentiate and communicate with each other in a way to maintain the homeostasis of a specific tissue or organ. However, cancer cells are also able to create a new and specific way to behave and communicate with each other and with the cells in their newly developed microenvironment. In this context, the microenvironment of normal tissue and the TME present various similarities and differences that result from the nature of their cellular interaction and the importance of the mediators and signaling pathways involved. In this review, we highlighted the importance of the dynamic reciprocity interaction at the single cell level, that involves the nucleus and cellular extracellular matrix elements. We also indicated how various secreted molecules and mediators are involved in the reciprocal cellular interaction and their role in the control of the fate of the developing tumors. Indeed, the tumor stroma provides exceptional organizational features where cancer cells respond to the constituents of the TME through modulation of the expression/activity of proteins involved in cell survival, proliferation and migration. On the other hand, tumor cell-derived signals activate and recruit various cells such immune cells, fibroblasts, adipocytes and endothelial cells that acquired cancer-associated phenotype and influence the structure and the composition of the TME by releasing ECM enzymes and components, exosomes, cytokines, and growth factors, all influence cancer cell functions and metabolism. Although these activated associated-cancer cells often enhance tumor growth and invasion, their activation can also repress tumor growth and invasion using the same released mediators. Thus, in a reciprocal manner, tumor cells influence the stroma and vice versa, jointly driving cancer progression or repression. The identification and the understanding of all these cancer causal factors and the degree of the importance of each reciprocal interaction in a temporal and geographical point of view will strongly help for the development of new prognostic and therapeutic strategies. This by taking in account not only the characteristic of the tumors and their TME but also the nature and the levels of the interactions between the signaling and the responding cells in the developing TME.

## Author Contributions

SP, SE, and A-MK wrote and drafted the manuscript. All authors contributed to the article and approved the submitted version.

## Funding

This work was supported by the Region Nouvelle Aquitaine, Ligue Contre le Cancer and Siric Brio.

## Conflict of Interest

The authors declare that the research was conducted in the absence of any commercial or financial relationships that could be construed as a potential conflict of interest.

## Publisher’s Note

All claims expressed in this article are solely those of the authors and do not necessarily represent those of their affiliated organizations, or those of the publisher, the editors and the reviewers. Any product that may be evaluated in this article, or claim that may be made by its manufacturer, is not guaranteed or endorsed by the publisher.
